# Nicotinamide N-Methyltransferase (NNMT): A New Hope for Treating Aging and Age-Related Conditions

**DOI:** 10.3390/metabo14060343

**Published:** 2024-06-19

**Authors:** Jing-Jing Li, Wei-Dong Sun, Xiao-Juan Zhu, Ya-Zhong Mei, Wen-Song Li, Jiang-Hua Li

**Affiliations:** Physical Education College, Jiangxi Normal University, Nanchang 330022, China; 17870363478@163.com (J.-J.L.); sunweidong0@outlook.com (W.-D.S.); zhxjhh@163.com (X.-J.Z.); 18479334568@163.com (Y.-Z.M.); 15070959590@163.com (W.-S.L.)

**Keywords:** aging-associated diseases, nicotinamide N-methyltransferase (NNMT), nicotinamide adenine dinucleotide (NAD^+^), homocysteine (Hcy), cancer, diabetes, cardiovascular diseases, neurodegenerative diseases

## Abstract

The complex process of aging leads to a gradual deterioration in the function of cells, tissues, and the entire organism, thereby increasing the risk of disease and death. Nicotinamide N-methyltransferase (NNMT) has attracted attention as a potential target for combating aging and its related pathologies. Studies have shown that NNMT activity increases over time, which is closely associated with the onset and progression of age-related diseases. NNMT uses S-adenosylmethionine (SAM) as a methyl donor to facilitate the methylation of nicotinamide (NAM), converting NAM into S-adenosyl-L-homocysteine (SAH) and methylnicotinamide (MNA). This enzymatic action depletes NAM, a precursor of nicotinamide adenine dinucleotide (NAD+), and generates SAH, a precursor of homocysteine (Hcy). The reduction in the NAD+ levels and the increase in the Hcy levels are considered important factors in the aging process and age-related diseases. The efficacy of RNA interference (RNAi) therapies and small-molecule inhibitors targeting NNMT demonstrates the potential of NNMT as a therapeutic target. Despite these advances, the exact mechanisms by which NNMT influences aging and age-related diseases remain unclear, and there is a lack of clinical trials involving NNMT inhibitors and RNAi drugs. Therefore, more in-depth research is needed to elucidate the precise functions of NNMT in aging and promote the development of targeted pharmaceutical interventions. This paper aims to explore the specific role of NNMT in aging, and to evaluate its potential as a therapeutic target.

## 1. Introduction

The aging process is marked by an intricate deterioration in the functionality of cells, tissues, and the entire organism, which heightens the risk of disease and mortality. To date, nine distinct biological pathways have been recognized as the key molecular mechanisms underlying aging. These include telomere shortening, cellular senescence, genomic instability, stem cell depletion, mitochondrial dysfunction, epigenetic changes, disrupted nutrient sensing, altered cell–cell communication, and decreased proteolysis [1]. With advancing age, individuals become increasingly prone to developing aging-related diseases, such as cancer, diabetes, cardiovascular conditions, and neurodegenerative disorders [2]. It is important to note that these diseases are not solely caused by aging itself, but rather by a combination of genetic, environmental, and lifestyle factors.

Nicotinamide N-methyltransferase (NNMT) is a pivotal enzyme with abundant expression in the liver and adipose tissue [3]. It facilitates the transfer of a methyl group from S-adenosylmethionine (SAM) to nicotinamide (NAM), yielding 1-methylnicotinamide (MNA) and S-adenosyl-L-homocysteine (SAH), the latter of which is promptly converted to homocysteine (Hcy) through hydrolysis [4]. As a consequence of consuming NAM (the precursor of NAD^+^) and producing SAH (the precursor of Hcy), NNMT plays a pivotal role in the NAD^+^-dependent signaling pathways and Hcy metabolism [5,6].

Authors have recently intensified their focus on NNMT’s function as a critical modulator of NAD^+^ and Hcy metabolic pathways, particularly in the context of age-related pathologies such as neurodegenerative disorders, diabetes, cardiovascular conditions, and cancers [7]. Several studies have demonstrated that NNMT expression and activity increase with age. For instance, the NNMT gene expression was significantly elevated in hepatocytes from senescent mice [8,9]. Furthermore, the treatment of aged mice with NNMT inhibitors activates senescent muscle stem cells and promotes muscle regeneration during the aging process [10]. It has also been suggested that NNMT may promote senescence, and that its expression is upregulated in senescence-related diseases. Kocinaj et al. [11] demonstrated that the NNMT expression was significantly increased in the neurons of patients with Alzheimer’s disease (AD). Ulanovskaya et al. [12] found that NNMT plays a significant role in governing the metabolic profile of cancer-associated fibroblasts (CAFs). Elevations in NNMT activity have been documented across various malignancies, including bladder, breast, colorectal, gastric, lung, oral cavity, ovarian, and prostate cancers, as well as in gliomas, lymphomas, and insulinomas [7,13,14]. Within these tumor types, NNMT has been implicated in enhancing the migratory, invasive, proliferative, and survival capabilities of cancer cells. Additionally, the NNMT levels have been found to be upregulated in the white adipose tissue (WAT) and livers of diabetic murine models, and the suppression of NNMT has been shown to guard against diet-induced obesity and insulin resistance [15]. Our previous studies also found genetic associations between NNMT gene and cardiovascular diseases [16,17]. A total of 308 individuals with primary hypertension and 395 with hyperlipidemia were enrolled from a cohort of unrelated Chinese Han individuals. A specific single-nucleotide polymorphism (SNP) located within the NNMT gene, rs1941404, was significantly linked to the occurrence of both hypertension [16] and hyperlipidemia [17]. This evidence indicates that modulating NNMT activity could potentially offer a viable therapeutic strategy for managing aging and its associated health issues. However, the exact regulatory mechanism of NNMT on aging remains unclear. This paper will review the current research on the relationship between NNMT and aging and aging-related diseases, as well as the potential mechanisms underlying this association. It will also present new ideas for targeting NNMT to prevent and control aging.

## 2. Potential Metabolic Pathways Linking NNMT to Aging

**Figure 1:**
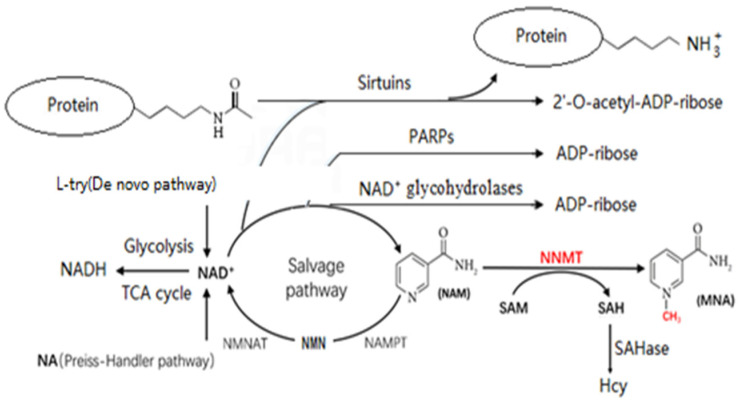
The metabolic pathways linking NNMT to NAD^+^ and Hcy. SAH, S-adenosyl-l-homocysteine; Hcy, homocysteine; NAD^+^, nicotinamide adenine dinucleotide; PARPs, poly-ADP-polymerases; NAD^+^, glycohydrolases, including CD38, CD157, and SARM1; L-try, L-Tryptophan; MNA, methylnicotinamide; NMN, nicotinamide mononucleotide; NA, nicotinic acid; NAM, nicotinamide; NNMT, nicotinamide N-methyl transferase; SAM, S-adenosyl-methionine; NAMPT, nicotinamide phosphoribosyltransferase; SAHase, S-adenosyl-homocysteine hydrolase; NMNAT, nicotinamide mononucleotide adenylyltransferase.

As illustrated in Figure 1, NNMT methylates NAM, resulting in the generation of SAH and MNA. This enzymatic process has the potential to deplete NAM, the precursor of nicotinamide adenine dinucleotide (NAD^+^), while simultaneously generating SAH, the precursor of Hcy. This ultimately leads to a decline in the NAD^+^ levels and an increase in the Hcy levels. Numerous studies have demonstrated that reduced NAD^+^ levels and elevated Hcy levels are pivotal factors in developing aging and age-related conditions. In this review, we outline the evidence available in the literature on the mechanisms by which NNMT activity results in decreased NAD+ levels and increased Hcy levels, and their correlation with aging and age-related disorders.

### 2.1. Consequences of Increased Homocysteine Levels

Souto et al. [18] identified NNMT as a key genetic factor influencing plasma Hcy concentrations through a genome-wide association study. As shown in Figure 1, NNMT methylates NAM, resulting in the formation of MNA and SAH. Subsequently, SAH is rapidly converted to Hcy by SAH hydrolase (SAHase). Therefore, increased NNMT activity leads to elevated blood Hcy levels.

Various investigations have shown a strong correlation between elevated homocysteine levels, known as hyperhomocysteinemia (HHcy), and the incidence of multiple diseases associated with aging, including cardiovascular diseases [19], neurodegenerative diseases [20], diabetes [21], and cancer [22]. For instance, Hcy has been widely reported to induce oxidative stress in cardiomyocytes by activating the oxidative system and inhibiting the antioxidant system [23]. Additionally, HHcy has been demonstrated to promote vascular inflammation through the activation of Toll-like receptor 4 (TLR-4). It has been shown that TLR-4 induces apoptosis, and that the mutation of TLR-4 attenuates the vascular inflammatory response and mitochondrial dysfunction in HHcy mice [24]. Moreover, increased concentrations of Hcy are recognized as a distinct risk factor contributing to the development of neurological conditions [25]. The evidence indicates that elevated blood Hcy levels can disrupt methylation or increase redox stress, leading to neurological imbalances and, subsequently, neurodegenerative disorders such as Parkinson’s disease [26], dementia [27], and attention deficit disorders [28]. Furthermore, higher plasma Hcy concentrations are found in individuals with type 2 diabetes, and these levels show a direct association with the severity of the insulin resistance and elevated glycosylated hemoglobin levels [29]. Soinio et al. [21] demonstrated that patients with type 2 diabetes exhibited higher blood Hcy levels, and that patients with diabetes and hyperhomocysteinemia (HHcy) exhibited more severe disorders of glucose–lipid metabolism than those with diabetes onset alone. It has been reported that the increase in Hcy-modified proteins promotes the development of intestinal cancer. In addition, the expressions of methylthionyl tRNA-synthetase (MetRS) and homocysteine-acylated protein tissues are significantly elevated in intestinal cancer in comparison with those in the surrounding normal tissues [30]. In conclusion, elevated homocysteine levels contribute to the development of aging and age-related diseases.

### 2.2. Consequences of Decreasing NAD+ Levels

NAD^+^ plays a crucial role in governing cellular energy metabolism, as well as the mitochondrial function and bioenergetic processes [31,32]. It acts as a hydride ion acceptor, transitioning to its reduced form, NADH, which is integral to the metabolic reactions across all organisms. NADH is particularly important for the activation of dehydrogenases, enzymes that facilitate key steps in various catabolic routes, such as glycolysis, the tricarboxylic acid (TCA) cycle, and fatty acid oxidation [33]. Additionally, NAD^+^ can be converted into its phosphorylated counterpart, NADP^+^, which serves as a precursor for NADPH. NADPH is essential for countering oxidative stress and facilitating fatty acid metabolism [33].

As illustrated in Figure 1, there are three distinct synthetic pathways for NAD^+^, namely, the Preiss–Handler pathway, the de novo pathway, and the salvage pathway (Salvage). The salvage pathway (Salvage) represents the primary source of NAD^+^ biosynthesis in mammals, accounting for over 85% of the NAD^+^ synthesis in mammals [34,35]. In this pathway, the synthesis of NAD^+^ from nicotinamide (NAM) is mediated by the enzymes nicotinamide phosphoribosyltransferase (NAMPT) and nicotinamide mononucleotide adenylyltransferase (NMNAT) [36]. Conversely, NAD^+^ can be broken down by a group of enzymes including poly (ADP-ribose) polymerases (PARPs), sirtuins, and NAD^+^ glycohydrolases, such as CD38, CD157, and SARM1, resulting in the production of NAM and ADP-ribose [37]. These NAD^+^-consuming enzymes play key roles in the cell signaling pathways that affect DNA repair, epigenetic modifications, circadian rhythms, inflammation, response to stress, energy metabolism, and lifespan [37]. Research has revealed that enzymes that utilize NAD^+^ compete for the limited pool of bioavailable NAD^+^, and that the concentrations of NAD^+^ significantly influence their functional activities. For example, the removal of PARP1 in mice led to elevated NAD^+^ levels and enhanced SIRT1 activity [38], whereas the absence of CD38 in mice resulted in a substantial rise in the NAD^+^ levels and increased sirtuin deacetylase activity [39]. These findings indicate that PARPs, CD38, and nuclear sirtuins draw from the same NAD^+^ pool. The inhibition of PARPs or CD38 has the potential to upregulate sirtuin activity. The augmented activation of sirtuins is increasingly viewed as a promising strategy for lifespan extension and the promotion of the healthspan. Conversely, the unregulated activation of PARPs and NAD^+^ glycohydrolases, such as CD38, may have detrimental effects, potentially exacerbating the aging process. Hence, while NAD^+^ glycohydrolases, sirtuins, and PARPs share the commonality of depleting NAD^+^, they also play unique roles in the aging process and the development of age-related diseases.

In the salvage pathway, NAM serves as a precursor for NAD^+^ synthesis [4]. NAM undergoes a dynamic cycle of synthesis, breakdown, and recycling within the cell to preserve stable levels of intracellular NAD^+^ [7]. Yet, with aging, this homeostatic balance is disrupted as the catabolic pathways gain prominence over the anabolic ones, potentially leading to a situation in which the breakdown of NAD^+^ outpaces the cell’s NAD^+^ synthesis capabilities. In contrast, the expression and activity of NNMT increase with age [8]. When NNMT is overexpressed, excess NAM is methylated by NNMT, resulting in a reduction in NAD^+^ synthesis [5]. This, in turn, affects the levels of NAD^+^ and the activities of NAD^+^-consuming enzymesv [40,41]. Consequently, it can be postulated that reduced NAD^+^ levels may be a key mechanism by which NNMT affects aging and age-related diseases.

## 3. Possible Mechanisms by Which NNMT Affects Aging through Hcy

As shown in Figure 1, NNMT methylates NAM to produce MNA and SAH, and then SAH is converted to Hcy by SAH hydrolase (SAHase). Therefore, increased NNMT activity leads to an increase in the blood Hcy levels. Studies have shown that elevated Hcy is significantly associated with several age-related diseases, including diabetes [29], cancer [42], hypertension [43], Alzheimer’s disease [44], and inflammation [45]. The following sections discuss the possible mechanisms by which NNMT affects aging through the Hcy pathway.

### 3.1. Hcy and Mitochondrial Dysfunction

The aging process and a host of age-related diseases are marked by compromised mitochondrial function. Mitochondria are central to maintaining cellular homeostasis and perform essential roles in oxidative phosphorylation, ion balance, redox regulation, lipid metabolism, metabolite production, cell differentiation, immune responses, and the anti-apoptotic and anti-aging pathways [46]. The accumulation of mitochondrial DNA (mtDNA) mutations is linked to mitochondrial impairment and the onset of aging-related pathologies such as neurological disorders, cardiovascular disease, and cancer [47]. As organisms age, the mitochondrial membrane’s loss of the thioredoxin–oxygen ATP complex, due to the opening of the mitochondrial permeability transition pore, contributes to progressive mitochondrial dysfunction [48].

Elevated levels of Hcy have been implicated in exacerbating mitochondrial dysfunction, which may be a mechanism by which Hcy influences aging. In rats with acute myocardial ischemia–reperfusion injury, high plasma Hcy levels have been shown to trigger mitochondrial dysfunction, potentially through increased cytochrome c release, enhanced reactive oxygen species (ROS) production, and extracellular signal-regulated kinase 1/2 (ERK1/2) signaling pathway activation, leading to cardiac impairment [49]. In rodent models, homocysteinemia can disrupt the mitochondrial function by reducing the levels of myotonic dystrophy protein, mitochondrial transcription factor A (mtTFA), and its regulator nuclear respiratory factor 1 (NRF-1) [50].

Hcy also inhibits the activity of mitochondrial complexes I–III in rats with ischemic brain injury, associated with increased cytochrome c release. Additionally, Hcy treatment in animals has been associated with elevated 8-hydroxy-2′-deoxyguanosine (8-OHdG) contents and mitochondrial Signal Transducer and Activator of Transcription 3 (Stat3) protein phosphorylation. Following focal cerebral ischemia, Hcy exacerbates the mitochondrial structural damage in the cerebral cortex and hippocampal dentate gyrus [51]. It impairs mitochondrial respiration, reduces electron transport chain (ETC) complex III activity, disrupts the mitochondrial membrane permeability, and induces apoptosis via the caspase-3 activation and calpain I-mediated pathways in umbilical vein endothelial cells [52]. In a rat model of Parkinson’s disease, Hcy decreased the mitochondrial complex I activity and induced oxidative stress in the substantia nigra, which was associated with increased hydroxyl radical production, reduced glutathione levels, and elevated antioxidant enzyme activity, such as superoxide dismutase and catalase [53].

### 3.2. Hcy and Cellular Aging

Cellular aging is defined as the irreversible loss of the cell replication ability and cell cycle arrest in the G0/G1 phase. Despite these changes, the cells retain a certain degree of metabolic activity [54]. Cellular aging is a hallmark of aging defined by a stable exit from the cell cycle in response to cellular damage and stress [55]. The study shows that the most reliable biomarker for determining that a cell is senescent may be the expression of the senescence-associated beta-galactosidase (SA-β-gal) enzyme. This enzyme is a biomarker that can be used to identify senescent cells both in vitro and in vivo. Other biomarkers that tend to persist in senescent cells are the accumulation of DNA damage-associated proteins, such as p16, p21, and p53, and the upregulation of cell cycle inhibitors, such as p16 and p21 [56]. The accumulation of senescent cells is a significant contributor to organ dysfunction, which is intricately linked to the emergence of numerous age-related illnesses, including cardiovascular diseases [57], neurodegenerative diseases [58], and others. These conditions have a profound impact on the well-being of patients.

HHcy has been recognized as a considerable contributor to the aging process at the cellular level [59]. The presence of elevated Hcy levels may accelerate this aging process, which, in turn, predisposes individuals to a range of age-related diseases. Hallmarks of cellular aging include the attrition of telomeres and a decline in telomerase activity [48]. Telomeres naturally shorten with each cycle of cell division, and their dysfunction can arise from the depletion of telomeric DNA sequences or the loss of protective telomeric proteins. This dysfunction triggers a DNA damage response, impairing cell proliferation and potentially leading to cellular senescence or apoptosis [60]. Zhang et al. [61] reported that exposing cultured endothelial cells to Hcy results in telomere attrition and an increase in acidic β-galactosidase, a biomarker of cellular aging. Additionally, Hcy has been observed to enhance the expression of cellular aging markers in these cells, such as p16, p21, and p53. Nevertheless, these effects can be alleviated by taking folic acid or SAM. Additionally, it has been demonstrated that Hcy induces endoplasmic reticulum stress in vascular endothelial cells, which, in turn, initiates apoptosis [62]. Elevated levels of Hcy can result in damage to the endoplasmic reticulum and redox abnormalities, which disrupt the disulfide bonds in folded proteins and form unfolded proteins [63]. These unfolded proteins can enter the nucleus and act as transcription factors, regulating the regulatory sequences of a variety of genes. This affects the transcriptional regulation of genes and the transcription of cytosolic cyclin A, inhibiting the proliferation of endothelial cells. Wang et al. [64] found that exposing human aortic endothelial cells to Hcy for 24 h led to an increase in the proportion of cells in the G1 phase and a reduction in the number of cells moving to the S phase. This indicates that Hcy inhibits cellular DNA synthesis and causes cell growth to stagnate in the G1 phase or at the transition point between the G1 phase and the S phase. This study additionally revealed that Hcy diminishes the activities of ERK1 and ERK2, isoforms of mitogen-activated protein kinase (MAPK), which is a crucial signaling system in living organisms and is involved in regulating cell growth, differentiation, and death, as well as intercellular functions [64]. Furthermore, it has been reported that Hcy selectively reduces the expression of cytokinin AmRNA and protein in human umbilical vein endothelial cells and affects cell proliferation [65]. Research into the neurotoxic effects of Hcy has revealed that it can trigger neuronal injury, which, in turn, activates N-methyl-d-aspartate (NMDA) receptors and results in cell death through processes such as excitotoxicity and apoptosis [66,67,68]. The effects of aging on enzyme activity, connective tissue, lipid synthesis, autoimmune disease, cardiovascular disease, and cancer are closely related to changes in Hcy metabolism [69].

### 3.3. Hcy and Oxidative Stress

Oxidative stress is a state in which the balance between the generation of reactive oxygen species (ROS) and the antioxidant defense system in the body is disrupted, resulting in the accumulation of ROS in the body and causing damage to cells and tissues [70]. The criteria for the assessment of oxidative stress include lipid peroxidation, protein carbonylation, oxysterols, and oxyphospholipids, which can effectively assess the level of oxidative stress in vivo and provide important references for the research and diagnosis of related diseases [71]. Oxidative stress is strongly associated with the onset and progression of many diseases, including neurodegenerative diseases, cardiovascular diseases, and cancers [72,73]. In addition, oxidative stress is thought to play an important role in the aging process.

Hcy at elevated levels has been associated with the aging process and an array of age-related conditions, including cognitive decline, attention deficits, and forms of dementia. It is well known that high Hcy levels are associated with oxidative stress [74], and that oxidative stress is a key factor in these diseases. Imbalances in the redox state and oxidative stress are thought to be central to the pathogenesis of HHcy. When Hcy binds to plasma proteins or other low-molecular-weight thiols, its free thiol group is oxidized, leading to the production of ROS. Proposed mechanisms for Hcy-induced oxidative stress include the au-to-oxidation of Hcy, the inhibition of antioxidant enzyme expression or activity, and the disruption of extracellular superoxide dismutase (SOD) on endothelial cell surfaces or NO synthase-dependent superoxide anion generation [75,76]. ROS generated by Hcy contribute to lipid peroxidation and cellular damage. Consequently, Hcy levels have been extensively studied in the context of oxidative stress, particularly in vascular cells and tissues, where elevated Hcy is considered a risk factor for cardiovascular disease [77]. Moreover, in young adults with central retinal vein occlusion, Hcy has been linked to oxidative stress [78]. Beyond its implications for cardiovascular health, altered homocysteine (Hcy) levels are associated with disruptions in the cellular redox balance and heightened oxidative stress. These conditions are concomitant with the production of ROS in various types of cells, such as neurons, endothelial cells, and glial cells, which can contribute to the onset of neurological conditions [79].

### 3.4. Hcy and Inflammation

Both animal and human studies have shown that Hcy is also a factor in inflammation. Elevated Hcy levels can impact various inflammatory markers, such as adhesion molecules, endothelial dysfunction, oxidative stress, white blood cell adhesion, and reduced nitric oxide (NO) availability [80]. This may lead to diseases associated with inflammation, including cardiovascular and neurological dysfunction [80,81,82]. Additionally, in patients with rheumatoid arthritis, high Hcy levels have been associated with an increased risk for cardiovascular disease and atherosclerosis. Yang et al. [83] reported increased Hcy levels, along with related immunoinflammatory and metabolic indicators, in individuals with rheumatoid arthritis. These findings suggest that these markers may be valuable in assessing the cardiovascular disease risk among patients with rheumatoid arthritis.

Furthermore, Hcy has been shown to induce inflammation in mouse retina, brain, and monocytes. Mild hyperhomocysteinemia has been linked to increased inflammatory cytokines in the brain in Wistar rats, including TNF-alpha, interleukin (IL)-1β, IL-6, and the chemokine monocyte chemotactic protein-1 (MCP-1) [82]. Treatment with Hcy was also observed to increase the pro-inflammatory cytokines and decrease the anti-inflammatory cytokines in a human retinal pigmented epithelial cell line (ARPE-19) [80]. In retinal endothelial cells exposed to Hcy, pro-inflammatory cytokines were detected [84].

What is more, elevated Hcy levels have been linked not only to cytokine-mediated inflammation but also to non-cytokine inflammatory mechanisms [85]. Specifically, Hcy has been shown to affect the expression and activity of matrix metalloproteinases (MMPs), which play a critical role in extracellular matrix remodeling and the progression of various inflammatory conditions [86]. Studies have shown that HHcy is correlated with an increase in extracellular matrix remodeling (ECM) via matrix metalloproteinases (MMPs) and the plasminogen/plasmin system. This results in an increased deposition of collagen that leads to endothelial–myocyte (EM) and myocyte–myocyte (MM) uncoupling; the physiological consequences are a plethora of cardiovascular pathologies [87].

In summary, the role of Hcy in mediating oxidative damage and inflammatory responses must be carefully evaluated in the context of cardiovascular disease, neurological dysfunction, and other age-related conditions.

## 4. Possible Mechanisms by Which NNMT Affects Aging through NAD^+^

NAD^+^, a critical coenzyme in redox reactions [33], is intricately involved in various cellular processes, such as energy production, DNA repair, chromatin modification, cellular aging, and epigenetic regulation. These functions are fundamental to sustaining the metabolic balance and promoting healthy aging. NAM serves as the primary precursor for NAD^+^ production. However, when NAM is methylated to form MNA by NNMT, it is no longer available for NAD^+^ synthesis, thereby potentially reducing the NAD^+^ levels. Research indicates that the NAD^+^ levels tend to decrease with age. Dysregulated NAD^+^ metabolism has been implicated in the etiology of numerous age-related conditions, including diabetes [88], cardiovascular disease [89], neurodegenerative diseases [90], and inflammation [8].

NAD^+^ also influences the expression of sirtuins, PARPs, and NAD^+^ glycohydrolases (CD38, CD157, and SARM1) (Figure 1), which are pivotal in the progression of age-related diseases. SIRT1, in particular, is instrumental in promoting insulin secretion in pancreatic β-cells in response to glucose [91] and has been shown to protect against insulin resistance in peripheral tissues such as adipose, liver, and skeletal muscle [92], indicating its significance in maintaining glucose homeostasis and preventing type 2 diabetes. CD38 has been identified as a central enzyme in inflammaging and aging, conditions that are closely linked to neurodegenerative diseases [93]. SARM1’s role in promoting axonal damage was established in an animal model, and mice lacking SARM1 are protected from axonal degeneration [94]. Furthermore, an in vivo study has shown that PARP1 activation is associated with the pathogeneses of Alzheimer’s disease [95] and Parkinson’s disease [96], indicating that PARP1 deficiency prevents brain dysfunction and cognitive decline. The following sections will discuss the potential mechanisms by which NNMT affects aging through the NAD^+^ pathway.

### 4.1. Decreased NAD^+^ Levels and Aging

The decline in the NAD^+^ levels with advancing age has been documented in various human tissues, including the liver [97], skin [98], brain [99], plasma [100], skeletal muscle [101], and monocyte-derived macrophages [102]. Moreover, NAD^+^ depletion is a hallmark of diseases characterized by accelerated aging [103,104]. Given the robust association between NAD^+^ activity and critical determinants of health and longevity [105], enhancing the NAD^+^ levels has the potential to mitigate or even reverse a variety of age-related conditions. 

As illustrated in Figure 1, NNMT regulates NAD^+^ biosynthesis by metabolizing NAM through the salvage pathway. A multitude of studies have demonstrated that the knockdown of NNMT in mouse adipocytes significantly elevates the intracellular NAD^+^ levels [15]. Silencing NNMT in a human colon cancer cell line (HT-29 cells) resulted in an approximate 30% increase in the NAD^+^ levels, while the overexpression of NNMT in a human colon cancer cell line (SW480 cells) led to a 30% decrease in the intracellular NAD^+^ levels [106]. The knockdown of NNMT in cancer-associated fibroblasts (CAFs) resulted in an increase in the NAD^+^ levels [107]. A study by Takahashi et al. [108] demonstrated increased renal NNMT expression and decreased NAD^+^ in a mouse model of unilateral ureteral obstruction (UUO). The results of Sun et al. [109] further indicated that in U251 glioma cells, the knockdown of NNMT in a low-glycemic environment increased the NAD/NADH ratio, while the opposite effect was observed in a high-glycemic environment. The NAD/NADH ratio is essential for maintaining intracellular NAD homeostasis. NNMT overexpression has been shown to decrease NAD^+^ and to lead to disruption in glucose metabolism [110]. The glucose intermediate metabolite a-ketoglutarate (a-KG) is a cofactor of methylcytosine hydroxylases (TET) and the JumonjiC demethylase family (JMJC HDM), which are involved in the demethylation of DNA and histones, respectively. Moreover, some glucose metabolites can enter the nucleus and influence the epigenetics [12]. Therefore, the decrease in the NAD^+^ levels may be a pivotal mechanism by which NNMT affects aging.

### 4.2. Sirtuins and Aging

Sirtuins are a group of enzymes that rely on NAD^+^ for their deacetylation activities. During the sirtuin-mediated deacetylation of lysine residues, NAD^+^ is converted to NAM and ADP-ribose (Figure 1) [111]. Sirtuins can regulate aging by altering the protein activity and stability through lysine deacetylation [112]. Sirtuins, which act as pivotal controllers of longevity across various organisms, are known to influence an array of biological processes. These include cell proliferation, life extension, gene regulation, metabolic pathways, telomere dynamics, chromosome organization, glycolysis, DNA maintenance, cellular aging, and oxidative stress [113,114,115,116]. Extensive research has revealed that sirtuins contribute to longevity in model organisms like yeast, worms, flies, and mice, and that they also alleviate various age-related conditions in mouse models such as type 2 diabetes, cancer, cardiovascular ailments, and neurodegenerative diseases [117,118,119]. Among the seven known intracellular sirtuins, SIRT1, an anti-aging protein, can inhibit apoptosis and retard the aging process by modulating genes involved in apoptosis, oxidative stress, and energy utilization [120]. It has been shown that supplementation with NAD^+^ and its precursors can partially activate SIRT1, elevate the intracellular NAD^+^ concentrations, and delay the onset of cellular senescence, which can help prevent age-related neurodegeneration, vascular stiffening, and renal pathologies [121,122,123]. Additionally, other sirtuins are involved in the regulation of cellular senescence, with early studies identifying Sir2 and related genes as key mediators of calorie restriction-induced lifespan extension in specific genetic contexts [124]. Chopra et al. [125] found that the pharmacological inhibition of SIRT2 slowed the accumulation of polyglutamine at the N-terminus of neurons in mice, resulting in neuroprotection and a prolonged lifespan. SIRT3, a major protein deacetylase in mitochondria, plays a crucial role in maintaining the mitochondrial function and reactive oxygen species (ROS) homeostasis by activating ATP synthase and regulating the mitochondrial NAD^+^ levels [104]. SIRT6 regulates the telomere stability and aging-associated inflammatory responses through the NF-κB signaling pathway, thereby prolonging the lifespan in mice [126].

NAM, the precursor to NAD^+^, acts as a potent inhibitor of sirtuin enzymes. NNMT plays a regulatory role in NAD^+^ biosynthesis by metabolizing NAM. Studies have demonstrated that NNMT is capable of inducing the expression and activity of sirtuins [31]. Estep et al. [127] were the first to identify a correlation between the expression and activity of NNMT and sirtuins, observing that caloric restriction in mice led to an upregulation of both NNMT and sirt1. Furthermore, Sirt1’s activity is known to enhance gluconeogenesis while inhibiting cholesterol synthesis and lipogenesis [128], suggesting that NNMT may play a role in regulating these processes by stabilizing the Sirt1 protein [129]. Liu et al. [130] reported that the overexpression of NNMT significantly enhanced the expression of SIRT1-3 in the human neuroblastoma cell line SH-SY5Y, which lacks endogenous NNMT expression, and that the silencing of SIRT3 using siRNA in NNMT-expressing SH-SY5Y cells resulted in a reduction in the complex I activity to levels similar to those in wild-type SH-SY5Y cells, as well as in a significant decrease in the cellular ATP content. These findings indicate that Sirt3 is a pivotal mediator of NNMT-induced complex I activity and ATP synthesis.

Furthermore, MNA has been shown to bind directly to sirtuins through an uncharacterized binding site, preventing their degradation and thereby maintaining the cellular levels of sirtuin proteins [131,132,133]. Parsons et al. [134] found that treating SH-SY5Y cells with MNA enhanced the complex I activity and ATP synthesis, as well as the stability of NADH dehydrogenase (ubiquinone) Fe-S protein 3 (NDUFS3), a subunit of mitochondrial complex I. This effect may be attributed to the interaction of MNA with sirtuins.

### 4.3. PARPs and Aging

PARPs are primarily located in the nuclei of a wide range of cells and are the predominant DNA repair enzymes in mammals. In contrast, NAD^+^ is the sole substrate for PARPs. ADP-ribosylation (ADPr), facilitated by PARPs, is a crucial mechanism in the cellular response to various stimuli, especially in the context of oxidative stress-induced DNA damage [135]. Oxidative DNA damage plays a substantial role in the development of age-related conditions, interfering with the expression of genes essential for DNA repair and cellular proliferation [98]. The human body possesses a multitude of physiological protection and repair systems, including the activation of the DNA deficiency sensor PARP1 and the deployment of natural endogenous antioxidant defenses to mitigate free radical-induced damage in order to maintain cellular homeostasis [136]. NAD^+^ is essential for the PARP-mediated repair of oxidative DNA damage. PARP1 gene knockdown leads to an elevation in the NAD^+^ levels, whereas PARP2 gene knockdown enhances SIRT1 expression by a mechanism that involves binding to and suppressing the SIRT1 gene promoter [137]. During the process of cellular senescence, the hyperactivation of PARPs leads to a substantial increase in the catabolism of NAD^+^. This decline in the NAD^+^ levels and rise in nicotinamide have the potential to significantly reduce SIRT1 deacetylase activity [138]. During DNA repair, ADP-ribose polymerizes onto nuclear proteins, such as histones and transcription factors, at sites of DNA strand breaks. Adequate levels of NAM prevent the degradation of PARPs, thereby facilitating DNA repair [139].

NNMT plays a pivotal role in regulating the efficient cycling of NAM to NAD^+^. The inhibition of PARPs by 2-PY prevents the regeneration of NAM required for NAD^+^ synthesis, thereby disrupting the balance between NAD^+^ depletion and synthesis (Figure 1), which, in turn, reduces the NAD^+^ levels [140]. SIRT1 and PARP1 exhibit reciprocal regulation through their competition for a shared co-substrate, NAD^+^, and through their modification of each other to regulate their catalytic activities [141]. Studies have underscored the critical role of PARPs in maintaining the NAD^+^ balance. For instance, mice fed with a high-fat diet experienced elevated NAD^+^ levels, increased SIRT1 activity, enhanced mitochondrial function, and resistance to insulin resistance when treated with PARP inhibitors or when PARP1 and PARP2 were absent [38,142]. A correlation was observed between PARP1 activation, reduced NAD^+^ levels, and SIRT1 activity inhibition in patients with pigmented keratoses, progeria-like aging, ataxia telangiectasia, and Cockayne syndrome [143]. Interestingly, the lifespan of mice with Cockayne syndrome was prolonged and the severity of symptoms associated with PARP1 overactivation was reduced following treatment with PARP1 inhibitors or NAD^+^ supplementation [144]. This suggests that the deleterious effects of PARP1 activation are mediated through imbalances in NAD^+^ homeostasis in response to widespread DNA damage and genotoxic stress. Additionally, PARPs have been found to be chronically activated in senescent worms and mice (in the liver or skeletal muscle), leading to the increased poly-ADP-ribosylation of cellular proteins [145]. In addition, the inhibition of PARP activity can delay cellular aging. For instance, NAD^+^ supplementation inhibited the PARP activity in astrocytes from patients and mice with non-alcoholic fatty liver disease (NAFLD), restored the glycolysis and mitochondrial function, and reduced oxidative stress [146,147]. D’Andrea et al. [148] additionally demonstrated that the overexpression of NNMT in mesenchymal cancer stem cells depleted the intracellular NAM, which enhanced the PARP1 activity and increased the cancer cell resistance to chemoradiotherapy. Collectively, NNMT and its metabolite 2-PY may influence cellular senescence by regulating the expression and activity of PARP proteins [149].

### 4.4. NAD^+^ Glycohydrolases and Aging

The pivotal catalytic function of NAD^+^ glycohydrolases, including CD38, CD157, and SARM1, is centered on the glycohydrolysis of NAD^+^. This enzymatic process entails the scission of the glycosidic bond within NAD^+^, leading to the formation of NAM and ADP-ribose [33]. Consequently, NAM serves not only as the predominant precursor for NAD^+^ biosynthesis but also as a direct product of NAD^+^ glycohydrolases. As such, the intracellular concentration of NAM directly influences the activity of these enzymes. NNMT converts NAM to MNA, thereby diminishing the availability of NAM and subsequently lowering the levels of NAD^+^. This process also influences the activity of NAD^+^ glycohydrolases.

As individuals age, the expression and activity of CD38, a pivotal NAD^+^-consuming enzyme, increase, contributing to the reduction in the NAD^+^ levels that are characteristic of aging [150]. CD38 is also involved in a base-exchange reaction, swapping NAD(P)^+^ from NAM for nicotinic acid adenine dinucleotide (phosphate) (NAAD(P)) under acidic conditions [151]. Importantly, cyclic ADP-ribose, NAAD(P), and ADP-ribose serve as significant Ca^2+^-mobilizing second messengers, indicating CD38′s pivotal role in activating Ca^2+^ signaling and influencing diverse cellular processes, including immune cell activation [152] and metabolic disorders [39]. For example, the dysfunction of CD38 has been associated with weakened immune responses, metabolic disorders, and behavioral changes in mice. CD38 serves as a key biomarker for human leukemia and myeloma, and it plays a direct role in the development of human immunodeficiency virus infection and chronic lymphocytic leukemia. Furthermore, CD38 regulates insulin secretion and the progression of diabetes [153,154]. Higher NAD^+^ levels in CD38-knockout (CD38KO) mice prevent obesity and metabolic syndrome [39]. It was shown that CD38 knockdown or treatment with CD38 inhibitors increases the NAD^+^ levels and ameliorates mitochondrial dysfunction and glucose intolerance in mice, leading to therapeutic interventions against Alzheimer’s disease, diabetes, and tumors [155,156,157,158]. 

The functional significance of the CD157 enzyme in cellular biology and the aging process has not been extensively elucidated. Nevertheless, emerging evidence indicates that CD157, akin to CD38, is upregulated in tissues undergoing aging [8], and it may contribute to age-related pathologies, including cancer [159].

The catalytic activity of SARM1 is contingent upon the Toll/Interleukin Receptor (TIR) structural domain, which was initially non-catalytic and primarily associated with protein–protein interactions [138]. Whether and to what extent NNMT regulates SARM1 under physiological conditions is unknown. However, SARM1 mediates NAD^+^ catabolism to generate NAM, and NNMT may regulate SARM1 by regulating NAD^+^ biosynthesis and thus SARM1. SARM1’s NAD^+^ catabolic activity has been demonstrated to be pivotal in axonal degeneration [94]. The protein is predominantly expressed in neurons and is involved in neuronal morphogenesis and inflammation [160]; it is also expressed in immune cells such as macrophages and T lymphocytes, influencing their functions [161]. Initially identified as a negative regulator of the innate immune response, SARM1 acts downstream of Toll-like receptor signaling through its interaction with TRIF, a TIR domain-containing adaptor protein that induces interferon-β [162]. More recently, SARM1 has been recognized as a crucial mediator of axon death signaling [163]. In axons, SARM1 triggers a metabolic crisis due to the rapid depletion of NAD^+^ [164], and the loss of the SARM1 function delays the axonal degeneration. NAD^+^ mediates the self-repression of this key neurodegenerative protein, offering a novel therapeutic target for the development of treatments for axonopathies, brain injury, and other neurodegenerative diseases [138].

## 5. Research Progress on NNMT in Aging-Related Diseases

The hallmarks of aging include the gradual loss of physiological integrity, leading to impaired function and increased susceptibility to death. The major manifestation of aging is the increased susceptibility to age-related diseases, such as cancer, diabetes, cardiovascular diseases, and neurodegenerative diseases [1]. NNMT plays an important role in aging. In our previous studies, significant associations were observed between NNMT gene polymorphisms and various age-related diseases, including diabetes [41] and cardiovascular diseases [16,17]. In Table 1, below, we mainly review the research progress on NNMT in these aging-related diseases.

### 5.1. NNMT and Cancer

The elevated expression of NNMT has been observed in diverse forms of cancer, including renal clear cell carcinoma [172], bladder cancer [173], gastric carcinoma [174], colorectal carcinoma [175], and oral squamous cell carcinoma [176]. This overexpression is a common feature in several types of cancer. Evidence indicates that NNMT overexpression is associated with an increase in cancer cell survival, proliferation, migration, and invasion. These effects may be related to the involvement of NNMT in apoptosis, autophagy, and energy metabolism [177]. Cui et al. [110] demonstrated that in esophageal squamous carcinoma (ESCC) cells, the knockdown of NNMT significantly increased the cell sensitivity to 5-fluorouracil (5-FU) by promoting apoptosis. This was accompanied by a decrease in the expression of glucose consumption-related enzymes and glycolysis-related enzymes, resulting in decreased glucose consumption and lactate production. Furthermore, the Warburg effect was inhibited, suggesting that high levels of NNMT increase the survival rate of cancer cells in the chemotherapy setting. Li et al. [178] demonstrated that 5-methylquinoline (5MQ), an NNMT inhibitor, effectively inhibited the proliferation of HeLa cells, suggesting that NNMT may be involved in cancer cell proliferation. Among the studies related to the NNMT regulation of cancer cell proliferation, those related to metabolism are more extensive [109]. Tomida et al. [179] demonstrated that in colorectal cancer, curcumin further reversed the cancer cell proliferation by inhibiting NNMT transcription and blocking the cell cycle progression. Hong et al. [133] showed that the knockdown of NNMT in the adipose tissue of healthy mice prevented diet-induced obesity, and that this effect was achieved by increasing the ubiquitination and stability of the SIRT1 protein. SIRT1 is involved in the Warburg effect, which is the preferential use of the glycolytic pathway by cancer cells to obtain energy and produce lactic acid even under aerobic conditions. This promotes tumor tissue proliferation and invasion [180]. NNMT may improve cancer cell survival and proliferation by enhancing the Warburg effect. Hanahan et al. [181] found that the reprogramming of energy metabolism is an emerging hallmark of tumors. Ulanovskaya et al. [182] conducted an untargeted metabolomic study that revealed an association between elevated NNMT levels and a reduced SAM/SAH ratio, increased MNA levels, and a decrease in histone methylation, which are linked to the altered expression of genes related to cancer. This research suggests that NNMT influences methylation by modulating the SAM and SAH levels rather than by the direct action of MNA [182]. A separate study emphasized NNMT’s role in SAM depletion and reduced methylation potential as pivotal mechanisms in the regulation of cancer-associated fibroblast (CAF) differentiation [107]. A proteomic study indicated that NNMT was overexpressed in the stroma associated with metastasis, and that gene knockdown restored the cell morphology to that of normal retinal fibroblasts. NNMT controls the expression of CAF markers and pro-tumorigenic cytokines by mediating genome-wide DNA methylation alterations and the hypomethylation of repressive chromatin markers [107]. Collectively, these findings indicate that NNMT expression is elevated in various cancers, and that its downregulation can normalize the stroma and diminish the metastatic and invasive capabilities of cancer cells.

### 5.2. NNMT and Diabetes

Diabetes is a condition characterized by metabolic dysfunction, which includes reduced glucose tolerance, elevated fasting blood sugar levels, insufficient insulin production, or the presence of insulin resistance [183,184]. Our previous study also found a significant association between one SNP variant (rs1941404) in the NNMT gene sequence and type 2 diabetes (T2D) in the Chinese Han population [165]. Yaguchi et al. [185] also reported that NNMT is a key factor in the development of T2D. Elevated levels of MNA have been detected in the urine [165] and sera [116] of patients with T2D, suggesting an increase in NNMT activity during the pathogenesis of T2D. Supporting evidence comes from several key studies. Kannt et al. [18] revealed that the NNMT expression was elevated in the WAT of individuals with insulin resistance or T2D. They also noted that the plasma MNA levels were closely linked to the NNMT expression in the WAT and the severity of insulin resistance. Hong et al. [129] further elucidated the role of NNMT in the onset of T2D. In cell culture experiments, they observed that NNMT knockdown significantly reduced the hepatocyte glucose production by 50%, whereas NNMT overexpression led to a substantial increase in the glucose output. In a mouse model, they showed that NNMT knockdown in the liver of C57BL6/J mice resulted in a notable decrease in the fasting blood glucose levels overnight.

Studies by Kraus et al. [15] and Hong et al. [129] have contributed to a further understanding of the mechanisms by which NNMT modulates glucose metabolism and its role in disease progression. Kraus et al. [15] demonstrated that reducing the NNMT expression in diet-induced obese mice enhanced their glucose tolerance and insulin sensitivity. Hong et al. [129] investigated the impact of NNMT on gluconeogenesis, finding that NNMT silencing in primary hepatocytes reduced the glucose output and downregulated the expression of key gluconeogenic enzymes, such as glucose-6-phosphatase (G6pc) and phosphoenolpyruvate carboxykinase 1 (Pck1). Conversely, NNMT overexpression led to increased glucose production and the upregulation of these enzymes. In a mouse model, NNMT-deficient mice displayed lower fasting blood glucose levels and a diminished capacity to convert pyruvate to glucose compared to the controls, indicating NNMT’s role as a positive regulator of hepatocyte gluconeogenesis.

Hong et al. [129] proposed that NNMT’s influence on glucose metabolism is mediated through its product, methylated nicotinamide (MNA), and involves Sirt1, a pivotal regulator in this process. They discovered that Sirt1 suppression in hepatocytes resulted in reduced glucose production and the downregulation of G6pc and Pck1 expression, and that Sirt1 overexpression could counteract the effects of NNMT knockdown on these gluconeogenic enzymes. This suggests that Sirt1 is crucial for NNMT’s regulatory effects on glucose metabolism.

Furthermore, Hong et al. [129] identified a correlation between Sirt1 protein expression and NNMT expression in hepatocytes. In vitro, NNMT overexpression increased the Sirt1 protein levels in primary hepatocytes, while NNMT knockdown decreased them. In vivo, NNMT-deficient mice exhibited reduced Sirt1 protein expression in the liver. These data indicate that both NNMT and its product MNA can elevate Sirt1 protein expression, and that their effects on glucose metabolism depend on Sirt1’s involvement [129].

### 5.3. NNMT and Cardiovascular Diseases

Cardiovascular disorders (CVDs) are currently the primary cause of death worldwide. A number of studies have demonstrated a strong association between NNMT activity and cardiovascular disease [40]. Our prior research established a notable correlation between a specific single-nucleotide polymorphism (SNP) in the NNMT gene, rs1941404, and the incidence of hypertension [16] and hyperlipidemia [17] among the Chinese Han population. Liu et al. [186] observed a substantial association between circulating MNA levels and coronary artery disease and left ventricular systolic dysfunction in Chinese patients [187]. Bubenek et al. [188] reported a strong association between NNMT expression, serum MNA levels, and the onset and progression of peripheral arterial occlusive disease. Interestingly, NNMT expression has been found to be positively associated with LDL levels and negatively associated with HDL levels, both of which are factors associated with cardiovascular disease [189,190,191].

MNA is an enzymatic reaction product of NNMT and has a natural inhibitory effect on it [192]. Swaminathan et al. [193] elucidated the crystal structure of the ternary complex of NNMT bound to MNA, illustrating that MNA occupies the active site on NNMT, impeding the binding of NAM and inhibiting the enzyme’s catalytic activity. Studies have identified MNA, a product of NNMT, as a potential therapeutic target for cardiovascular conditions such as thrombosis, hypertension, and atherosclerosis [194,195,196]. MNA has been shown to reverse endothelial dysfunction by enhancing nitric oxide (NO) production, thereby offering protection to the aorta [197]. In an animal model of hypercholesterolemia (ApoE/LDLR-/- mice), MNA was found to increase the Tyr/Phe ratio and improve the endothelial function [198]. Additionally, MNA promotes prostacyclin (PGI2) secretion by endothelial cells, preventing platelet aggregation, smooth muscle cell proliferation, and leukocyte adhesion to the endothelium [199,200]. MNA also demonstrates anti-inflammatory effects and enhances the endothelial NO and PGI2 levels, as evidenced by studies indicating that MNA administration does not diminish the reduction in inflammatory cytokines in macrophages [201].

Additionally, it has been demonstrated that treatment with methylated nicotinamide (MNA) and nicotinamide (NAM) can significantly reduce the atherosclerotic plaque size, decrease macrophage infiltration, and lower the levels of lipids such as cholesterol, cholesteryl esters, and triglycerides [171]. It is worth noting that the conversion of NAM to MNA has been demonstrated to confer anti-inflammatory and cardioprotective properties [202,203]. In ApoE/LDLR-/- mice, MNA administration was found to have a more pronounced effect on reducing the atherosclerotic plaque formation compared to NAM. This may be attributed to the depletion of SAM, which could influence the epigenetics of endothelial cells [7].

In summary, these studies suggest that MNA exerts a positive effect on the endothelial function. However, the increased methylation activity of NNMT, which converts NAM to MNA, can inhibit NAM from entering the NAD^+^ salvage pathway, leading to decreased NAD^+^ levels. This, subsequently, may exacerbate the adverse effects associated with NAD^+^ depletion [204]. Consequently, the potential adverse effects of NNMT inhibitors on the endothelium should be thoroughly assessed when considering their use in the treatment of various age-related conditions [205].

In addition, oxidative stress contributes to the development of cardiovascular disease by damaging endothelial cells, promoting smooth muscle cell proliferation and migration, and inducing monocytes to adhere to the stem endothelium and differentiate into macrophages [206]. It has been found that members of the Sirtuins family play an anti-atherosclerotic role by reducing oxidative stress and preventing damage to endothelial cells, vascular smooth muscle cells, and macrophages, thereby reducing vascular injury [207]. UNGVAR et al. [208] demonstrated that SIRT1 overexpression significantly upregulated the expression of superoxide dismutase (MnSOD/SOD2), increased the content of reduced glutathione (GSH), inhibited the production of ROS, and lowered the cellular level of H2O2 in human coronary artery endothelial cells, which, in turn, exerted a protective effect on the endothelial damage induced by hyperglycemia and thereby inhibited the development of atherosclerosis. 

β-Hydroxybutyrate (β-OHB), a ketone body derived from fatty acid oxidation, is currently not only considered as an energy substrate to maintain metabolic homeostasis but also acts as a signaling molecule to regulate lipolysis and oxidative stress [209]. Several studies have shown that both Sirt1 and β-OHB are involved in the cellular antioxidant response [210]. β-OHB seems to work as a mimetic of caloric restriction, which is the most known natural activator of some sirtuins [210]. Edwards et al. [211] demonstrated that β-OHB administration in C. elegans delayed the glucose toxicity and extended the worm’s lifespan in a Sir2 (the homolog of the human Sirt1)-dependent manner. Therefore, these authors proposed β-OHB as a valuable treatment against aging-associated disorders [211].

### 5.4. NNMT and Neurodegenerative Diseases

Neurodegenerative diseases are a pressing health concern for the elderly. Research has revealed a significant association between the SNP rs694539 in the NNMT gene and neurodegenerative disorders such as bipolar disorder [168], schizophrenia [169], and epilepsy [170]. 

NNMT is exclusively expressed in neurons and exhibits varying levels of expression across different brain regions [167]. Schmeisser et al. [212] initially explored the role of NNMT in neuronal homeostasis, behavior, neurodegeneration, and lifespan using the model organism Caenorhabditis elegans. They found that neurons expressing a mildly ectopic form of anmt-1, the C. elegans homolog of human NNMT, regulate neurotransmitter production and neuronal autophagy by altering the availability of intracellular methyl groups. ANMT-1 competes with LCMT-1, the worm homolog of human LCMT1 (leucine carboxymethyltransferase 1), for the methyl group of SAM. Since the methylation to MNA is irreversible and initiates methylation priming in cells, this competition restricts the LCMT-1 activity. This, in turn, affects the pathway involving LET-92/PP2A (protein phosphatase 2) and NPRL-2/NPRL2 (a subunit of the human npr2-like GATOR1 complex), leading to the induction of autophagy. Thus, ANMT-1 impacts the neurohomeostasis, behavior, degeneration, overall health, and lifespan in C. elegans [212].

Parkinson’s disease (PD) is a prevalent neurodegenerative condition, and there is a notable upregulation of the NNMT expression in the brains of individuals who pass away from PD [167,213]. In vitro studies have shown that the overexpression of NNMT in the SH-SY5Y human neuroblastoma cell line confers various cytoprotective effects, such as increased complex I activity and ATP synthesis and resistance to mitotoxins linked to PD, including rotenone, 1-methyl-4-phenylpyridinium ion, and 6-hydroxydopamine [134,214]. One potential mechanism for the beneficial effects of increased NNMT expression is the activation of the Akt signaling pathway. Akt, a kinase critical for cell survival, has been implicated in the pathogeneses of neurodegenerative diseases, including PD and Alzheimer’s disease (AD), when its regulation is disrupted [166]. Akt phosphorylates downstream targets, such as glycogen synthase kinase-3β (GSK-3β), which, in turn, influences the tau protein phosphorylation [44]. In AD, Akt pathway downregulation is associated with GSK-3β overactivity, leading to tau hyperphosphorylation [215]. Studies have suggested that NNMT can activate the Akt pathway through ephrin-B2 [216]. Therefore, Bhalla and colleagues hypothesized that the elevated NNMT expression observed in AD patients may be a neuronal response to the stress caused by the disease’s pathogenic processes [217].

## 6. NNMT as a Therapeutic Target

If heightened NNMT expression contributes to the development of aging and age-related conditions, then inhibitors of NNMT could potentially alleviate these issues. The demonstration that the administration of antisense oligonucleotides targeting NNMT to animals led to reduced weight gain and enhanced insulin sensitivity has prompted the development of NNMT inhibitors (NNMTis) for the treatment of obesity and type 2 diabetes. Two distinct studies have shown the effectiveness of two different NNMTi compounds in treating obesity and related metabolic disorders in preclinical animal models. Kannt et al. [218] reported that the treatment of mice with a nicotinamide analogue inhibitor of NNMT (JBSNF-00008) reduced the body weight, improved the insulin sensitivity, and normalized the glucose tolerance in mice with high-fat-diet-induced obesity. Similarly, Neelakantan et al. [204] found that the systemic treatment of diet-induced obese mice with a quinoline-based NNMTi reduced the body weight and WAT mass while enhancing the plasma lipid profile. As observed in NNMT knockdown experiments, NNMTis counteract obesity by influencing NAD^+^ rescue and SAM-related pathways. These findings suggest that NNMT inhibitors could be combined with other dietary supplements, such as NAD^+^ precursors, to enhance the therapeutic effect of the supplements and minimize the side effects of high pharmacological doses.

Considering its documented impact on tumor progression, clinical outcomes, and drug sensitivity, NNMT presents a promising target for drug development in various cancers with altered NNMT activity. A recent study [204] investigated the use of NNMTis, which are typically employed for the treatment of diet-induced obesity in heart failure, in an intraperitoneal orthotopic model of ovarian cancer metastasis. The findings indicated that the NNMTis induced a reduction in the tumor burden and tumor cell proliferation and an increase in the stromal H3K27 trimethylation [107]. Eckert et al. [12] reported that NNMT contributes to tumorigenesis by regulating the methyl donor homeostasis within cancer cells. Therefore, NNMTis may hold therapeutic potential by influencing the cancer epigenome.

Neelakantan et al. [10] were the first to show that the systemic administration of a small-molecule analog of NNMT inhibitors to aged animals rejuvenated their muscle stem cells (muSCs) and restored the regenerative capacity within their aging skeletal muscle. Additionally, they reinstated the intrinsic static properties of muSCs to facilitate dynamic muscle repair mechanisms following injury. Importantly, NNMT inhibition promoted the regeneration and growth of injured muscle fibers, significantly enhancing the strength of the affected muscle, indicating an improvement in the overall function of the aging skeletal muscle. These findings suggest that NNMT inhibitors could be utilized as novel therapies to reinvigorate healthy myogenic responses in injured aging skeletal muscle by reviving the muSC activity, potentially mitigating the functional decline commonly experienced by older adults and enhancing the muscle performance following injury.

These results suggest that NNMT may be an enticing target for the development of oligonucleotide-based therapeutics to combat aging and age-related ailments. Nevertheless, oligonucleotide treatments encounter several technical challenges. These include ensuring the effective delivery of oligonucleotides to specific organs or tissues, overcoming interactions between oligonucleotides and their targets [219,220], and managing the sequence- and chemical-induced toxicity and the saturation of endogenous RNA-processing pathways [221]. Consequently, although oligonucleotide products targeting NNMT have demonstrated efficacy in laboratory and animal models, none have entered clinical trials to date. It is worth noting, however, that significant strides have been made in the potency and selectivity of small-molecule inhibitors of NNMT. The clinical relevance of NNMT in a range of conditions, such as cancer and metabolic disorders, has expedited the development of potent compounds targeting NNMT. Although these compounds are still in the early stages of preclinical development, this progress indicates that the obstacles to oligonucleotide therapy are being addressed, and that the development of oligonucleotide drugs targeting NNMT remains a promising avenue for future treatments of aging and age-related diseases.

## 7. Prospects for Future Research

As previously described, NNMT methylates NAM, which has a direct impact on cellular methylation homeostasis and NAD^+^ metabolism. Both SAM and NAD^+^ are important energy-metabolizing substances. Cells maintain their methylation balance by controlling the levels of SAM and SAH, which, in turn, regulate enzymes like protein methyltransferases and DNA methyltransferases. These enzymes are crucial for modulating histone methylation and gene expression [222]. NAM serves as the precursor for NAD^+^, which is integral to energy metabolism, encompassing processes like glycolysis, the tricarboxylic acid cycle, fatty acid oxidation, and alcohol metabolism. NAD^+^ is also a co-substrate for a variety of enzymes, including sirtuins [37]. Moreover, NAD^+^ functions as a nucleotide analog during DNA ligation and RNA sequestration [223]. Given that SAM is the primary methyl donor for protein, nucleotide, and lipid methylation, the overexpression of NNMT creates a metabolic sink for methyl groups. This sink depletes the available SAM, thereby potentially compromising genomic methylation processes [12]. Furthermore, NNMT catalyzes the conversion of NAM into MNA, which effectively sequesters NAM and prevents its participation in the NAD^+^ salvage pathway. Consequently, this enzymatic activity restricts the availability of NAD^+^, thereby constraining NAD^+^-dependent processes that rely on the post-synthetic modification of critical biomolecules [33]. The impact of NNMT on the SAM methylation equilibrium or NAD^+^ metabolism is contingent upon the tissue type and pathological scenario, which are typically associated with increased metabolic demands. It is apparent that NNMT is significantly expressed in metabolically active tissues like livers and adipose tissues, as well as in conditions involving heightened metabolic requirements, such as aging and age-related diseases, including cancer and diabetes. Despite the accumulation of data on how various transcription factors regulate NNMT expression in a tissue-specific manner, the precise mechanisms by which these regulations translate into coordinated tissue-specific gene regulation are yet to be fully understood. Although NNMT does not directly generate epigenetic enzyme cofactors, the reactions it catalyzes can alter the availability of key epigenetic enzyme cofactors (SAM and NAD^+^) or inhibitors (MNA and SAH). This could account for why alterations in NNMT lead to specific gene regulatory outcomes. Nevertheless, further research is necessary to uncover the precise mechanisms by which NNMT influences cell-type-specific gene expression. 

The impact of NNMT on anaerobic energy metabolism remains to be fully elucidated. While numerous studies have documented that NNMT inhibits the aerobic metabolism of sugars and lipids, with NNMT knockdown leading to increased oxygen consumption, enhanced sugar utilization, and reduced fat synthesis [224], the specific effects of NNMT on anaerobic energy metabolism have not been extensively investigated. Our previous research [225] revealed elevated NNMT expression in toe extensor muscles, which predominantly rely on anaerobic energy metabolism for force production, compared to that in flounder muscles. Furthermore, the inhibition of NNMT using MNA was associated with a decline in the endurance-free exercise performance in rats [226]. These findings suggest the possibility that NNMT may also play a role in regulating anaerobic energy metabolism.

With the aging of muscle tissue, there is an increase in NNMT expression, which correlates with a decline in the NAD^+^ levels. Building upon this discovery, NNMT inhibitors were successfully utilized to enhance the muscle regeneration in an aged mouse model of muscle injury [227]. Studies have shown that by manipulating the NAD^+^ salvage pathway, NNMT inhibitors can impact the intracellular NAD^+^ levels. This, subsequently, modulates the deacetylation of proteins by sirtuins, initiating cellular metabolic and transcriptional responses that promote the differentiation of adult myoblasts [10]. However, the potential epigenetic regulatory role of NNMT inhibitors in mitigating the aging-related decline in skeletal muscle, as well as the long-term effects of NNMT inhibitor treatment on the muscle functional performance, remain to be fully explored.

## 8. Conclusions

NNMT overexpression disrupts the balance of multiple metabolic pathways by reducing the NAD^+^ levels and increasing the Hcy levels, which accelerates aging and contributes to the development of age-related diseases. However, the exact mechanisms behind these phenomena remain unclear. Although the use of various oligonucleotide-based drugs and small-molecule inhibitors of NNMT has corroborated its potential as a therapeutic target for addressing aging and age-related diseases, their therapeutic efficacy has been impeded by issues such as low selectivity, poor metabolic stability, and suboptimal cellular permeability. As a result, further investigation is necessary to fully understand the role and mechanism of NNMT in aging and age-related diseases, and to develop more effective drugs targeting NNMT for the treatment and prevention of these conditions.

## Figures and Tables

**Table 1:** NNMT and age-related diseases.

Type of Disease	Specific Diseases	Correlation	Reference
Diabetes	Type 2 diabetes	NNMT Active ↑; The rs1941404 variant of the NNMT gene is significantly associated with T2D	[165]
Neurodegenerative Diseases	Alzheimer’s disease	NNMT expression ↑	[166]
	Parkinson’s disease		[167]
	Dementia		[27]
	Attention deficit disorder		[28]
	Bipolar disorder	The rs694539 variant of the NNMT gene is significantly associated with bipolar disorder	[168]
	Schizophrenia	The rs694539 variant of the NNMT gene is significantly associated with schizophrenia	[169]
	Epilepsy	The rs694539 variant of the NNMT gene is significantly associated with epilepsy	[170]
Cancer	Bladder cancer	NNMT Active ↑; Migration, invasion, proliferation, and viability of cancer cells ↑	[7,13,14]
	Breast cancer		[7,13,14]
	Colorectal cancer		[7,13,14]
	Gastric cancer		[7,13,14]
	Lung cancer		[7,13,14]
	Oral cavity cancer		[7,13,14]
	Ovarian cancer		[7,13,14]
	Prostate cancers		[7,13,14]
	Glioma cancer		[7,13,14]
	Lymphomas		[7,13,14]
	Insulinomas		[7,13,14]
	Esophageal squamous carcinoma	NNMT Active ↑; survival of cancer cells in the chemotherapy setting ↑	[110]
	Mesenchymal cancer	Overexpression of NNMT makes cancer cells resistant to chemoradiotherapy ↑	[148]
Cardiovascular Diseases	Hypertension	The rs1941404 variant of the NNMT gene is significantly associated with hypertension	[16]
	Hyperlipidemia	The rs1941404 variant of the NNMT gene is significantly associated with hyperlipidemia	[17]
	Atherosclerosis	MNA, a product of NNMT, is effective at reducing atherosclerotic plaque formation	[171]
Other Diseases	Sarcopenia	NNMT activates senescent muscle stem cells and improves the regeneration of ageing skeletal muscles	[10]

“↑” Indicates an increase in activity.

## Data Availability

Not applicable.
